# STARD3NL as a prognostic marker related to an immunosuppressive tumor microenvironment in hepatocellular carcinoma

**DOI:** 10.3389/fonc.2026.1911730

**Published:** 2026-08-05

**Authors:** Di Li, Ziyu Liu, Li Xie, Rui Li, Guocui Yang, Suyun Li, Xiaohan Qin, Jinping Lin

**Affiliations:** 1 New World Institute of Biotechnology, School of Biotechnology, East China University of Science and Technology, Shanghai, China; 2 Discipline Construction Department, National Engineering Research Center for Biochip at Shanghai, Shanghai, China; 3 School of Integrative Medicine, Shanghai University of Traditional Chinese Medicine, Shanghai, China

**Keywords:** hepatocellular carcinoma, multiplex immunohistochemistry, prognostic biomarker, STARD3NL, tumor microenvironment

## Abstract

**Background:**

Changes in the tumor microenvironment (TME) are closely involved in hepatocellular carcinoma (HCC) development, and lipid metabolic disorders may take part in this process. STARD3NL is known to be related to cholesterol trafficking and lipid homeostasis. However, little is known about whether STARD3NL is linked to immune features of the HCC microenvironment.

**Methods:**

We first explored STARD3NL expression and its prognostic relevance in HCC using The Cancer Genome Atlas (TCGA). To verify the bioinformatic findings, HCC tissue microarrays (TMAs) were analyzed by immunohistochemistry (IHC) for STARD3NL localization and survival evaluation. Multiplex immunohistochemistry (mIHC), combined with digital image analysis, was further used to characterize immune cell infiltration. Clinicopathological differences between tumors with high and low STARD3NL expression were also examined. In addition, spatial distance analysis was performed to assess how immune cells were arranged in STARD3NL-high regions.

**Results:**

TCGA-based analysis and mIHC validation both showed higher STARD3NL expression in HCC tissues. Patients with increased STARD3NL expression had poorer survival (p = 0.029), and multivariable analysis supported STARD3NL as an independent risk factor. STARD3NL-high tumors showed increased proportions of CD68+ macrophages and FOXP3+ T cells (p < 0.005). Spatial analysis further indicated that Tregs were more frequently located near macrophages (p = 0.001), with a shorter mean macrophage-Treg distance (p = 0.027). This macrophage-Treg proximity was associated with worse prognosis (p = 0.030).

**Conclusion:**

High STARD3NL expression is significantly associated with immune-suppressive features and unfavorable outcomes in HCC. These findings indicate that STARD3NL may have value as a prognostic biomarker associated with an immunosuppressive tumor microenvironment.

## 1 Introduction

HCC remains a highly aggressive malignancy and is a major cause of cancer-related death worldwide. According to recent data from the National Cancer Center of China, primary liver cancer ranked fifth for incidence and second for mortality in China in 2022 (1, 2). HCC accounts for about 75%-85% of primary liver cancers (3, 4). Despite improvements in surveillance and treatment, the clinical outcome of HCC is still unsatisfactory, largely because metastasis is common and many tumors respond poorly to conventional therapies.

Tumor-related inflammation has recently become an important focus in HCC research. Both systemic and local inflammatory changes may help predict recurrence and survival. Systemic inflammation can favor HCC progression by reducing apoptosis and increasing angiogenesis; for example, the systemic immune-inflammation index and the neutrophil-to-lymphocyte ratio have been linked to recurrence risk (5, 6). Long-standing inflammation also contributes to a complex and heterogeneous TME (7). Within this microenvironment, immune and stromal cells interact directly with tumor cells and influence tumor growth, metastasis, and immune escape (8–10). These cells include M1-like and M2-like macrophages, regulatory T cells (Tregs), and cancer-associated fibroblasts (CAFs). Antitumor M1-like macrophage activity is often impaired, whereas M2-like macrophages may promote tumor progression through immune-suppressive functions (11, 12). Tregs further support immune evasion (13), and CAFs can accelerate tumor growth through the secretion of growth factors (14).

Abnormal lipid metabolism is another factor that may shape immune suppression in the TME (15–17), especially through cholesterol-related pathways (18). STARD3NL is a late-endosomal membrane-anchored protein that forms membrane contact sites (MCSs) between late endosomes and the endoplasmic reticulum (ER) via interaction with ER-resident VAP proteins (19). Through this function, STARD3NL participates in intracellular cholesterol transport and lipid homeostasis (20). Cholesterol metabolism has been increasingly recognized as a critical determinant of immune cell function in turn: cholesterol accumulation can promote M2-like macrophage polarization (21), and lipid droplet-rich macrophages in HCC have been reported to recruit regulatory T cells (Tregs) via the CCL20/CCR6 axis, thereby establishing an immune-suppressive niche (22). Based on this background, we hypothesized that STARD3NL, through its role in cholesterol trafficking and lipid homeostasis, might be linked to the immune landscape of HCC.

STARD3NL has previously been linked to poor prognosis in gastric cancer (23), but its relevance in liver cancer and its connection with the TME remain insufficiently defined.

Here, we investigated whether STARD3NL expression is related to HCC prognosis and to immune features of the TME, with particular attention to immune cell phenotype and spatial distribution. Our results point to the conclusion that STARD3NL-high tumors are associated with features of immune suppression and worse clinical outcomes.

## 2 Materials and methods

### 2.1 Gene expression data analysis

We analyzed the TCGA liver hepatocellular carcinoma (LIHC) cohort together with the GEO dataset GSE76427. The GSE76427 expression matrix, generated on the Illumina platform, was normalized before tumor and paired adjacent normal tissues were compared with the R limma package (version 4.2.1; https://www.r-project.org/) (24). For RNA-sequencing data from TCGA-LIHC, the edgeR package in R was used for normalization and DEG screening (25). Upregulated genes shared by the two datasets were identified and visualized with the Venn Diagram package.

### 2.2 Survival-associated analysis

Kaplan-Meier survival curves were generated using TCGA data and the Kaplan-Meier Plotter database (https://kmplot.com/). For continuous variables used in survival analysis, optimal cutoffs were determined with Survminer and then applied to divide patients into high- and low-expression groups. Cox proportional hazards models were used to estimate the relationship between candidate variables and event risk over time. Hazard ratios (HRs) and p-values were reported. HR values above 1 were interpreted as indicating higher risk with increased gene expression, and p < 0.05 was used as the threshold for statistical significance.

### 2.3 Gene set enrichment analysis

To identify biological pathways associated with STARD3NL expression in hepatocellular carcinoma, we stratified patients from The Cancer Genome Atlas (TCGA) cohort into high- and low-STARD3NL expression groups based on the optimal cutpoint determined by the surv_cutpoint function in R, as described in Section 2.2. Differential expression analysis between the two groups was performed using the limma package, and all genes were ranked by log2 fold change to generate a pre-ranked gene list.

GSEA was conducted using the clusterProfiler R package (version 4.4.4) with gene sets derived from the Canonical Pathways (C2) collection of the Molecular Signatures Database (MSigDB, version 2026.1.Hs), which comprises curated signaling and metabolic pathways from databases such as KEGG, Reactome, and BioCarta. Enrichment scores were calculated using a weighted Kolmogorov-Smirnov statistic with 1,000 gene set permutations (26). Pathways with |normalized enrichment score (NES)| > 1.3, pvalue<0.05 and FDR q-value < 0.25 were considered significantly enriched.

### 2.4 Co-expression network construction and hub gene selection

Pearson correlation coefficients were calculated between gene pairs across samples to build the co-expression network. Gene pairs with R > 0.6 or R < -0.6 and p < 0.05 were retained. Cytoscape (version 3.9.1) was used for network visualization. The NetworkAnalyzer plugin was then applied to calculate topological features, including gene connectivity, and to identify hub genes.

### 2.5 scRNA-seq data processing

Single-cell RNA sequencing count matrices were downloaded from GEO. The Seurat R package (version 4.0.5) was used for quality control, normalization, dimensionality reduction, and clustering. Cells were removed if mitochondrial UMI proportions were greater than 25% or if fewer than 200 genes were detected. Principal component analysis was performed before clustering. FindNeighbors, FindClusters, and RunTSNE were used for cluster construction and visualization. Cluster marker genes were detected with FindAllMarkers, cell identities were assigned with SingleR, and dot plots were prepared using ggplot2.

### 2.6 Immune correlation prediction

The TIMER 3.0 platform, which integrates 15 state-of-the-art immune deconvolution algorithms (http://timer.cistrome.org/) (27) was used to evaluate the relationship between STARD3NL mRNA level and immune infiltration in LIHC.

### 2.7 Patients and tissue samples

This study included 158 HCC patients. TMAs named HLiv-HCC180Sur-04 and HLivH090Su01 were provided by the National Engineering Center for Biochip (Shanghai, Outdo Biotech). These arrays contained HCC tissues, matched adjacent non-cancerous tissues, and corresponding clinical information collected under applicable regulations. The study complied with the Declaration of Helsinki.

### 2.8 Immunohistochemistry and evaluation

TMA sections were baked at 63 °C for 1 h, deparaffinized using a LEICA ST5020 automated stainer, and rehydrated through graded ethanol. Heat-induced antigen retrieval was performed using a Dako PT Link system. After washing with PBS, the sections were incubated overnight at 4 °C with an anti-STARD3NL antibody (1:1500, 20502-1-AP, Proteintech). Blocking, secondary-antibody incubation, and DAB chromogenic detection were performed using a Dako Autostainer Link 48 and the EnVision FLEX+ detection system (K8002, Dako). The sections were counterstained with hematoxylin, mounted with neutral resin, and scanned using an Aperio XT scanner.

STARD3NL staining was evaluated independently by two pathologists who were blinded to the clinical information and survival outcomes. Staining intensity was scored as 0 (absent), 1 (weak), 2 (moderate), or 3 (strong), and the percentage of positive tumor cells was expressed as a proportion ranging from 0 to 1. A semiquantitative IHC score was calculated by multiplying the staining-intensity score by the positive-cell proportion, yielding a final score ranging from 0 to 3. Discordant cases were independently reviewed by a third pathologist to determine the final score. The optimal cutoff was determined using the survminer package. Tumors with an IHC score >1.485 were classified as STARD3NL-high, whereas those with a score ≤1.485 were classified as STARD3NL-low.

### 2.9 Immunofluorescence staining and image analysis

mIHC staining followed a published protocol (28) that had been validated by the Earle A. Chiles Research Institute IHC Core at the Providence Cancer Institute (Portland, OR). TMA slides were baked at 63 °C for 1 h, deparaffinized, subjected to antigen retrieval, and blocked. Before multiplex staining, each antibody was tested by single-plex immunofluorescence to confirm its staining localization and optimize the working concentration. Multiplex staining was performed sequentially as follows: STARD3NL (1:500, 20502-1-AP, Proteintech)–Opal 520; CD163 (prediluted, PA059, abcarta)–Opal 650; FAP (1:200, ab207178, Abcam)–Opal 570; FOXP3 (prediluted, PA448, abcarta)–Opal 690; CD68 (prediluted, PA014, abcarta)–Opal 620; and PanCK (prediluted, PA125, abcarta)–Opal 480. During each cycle, the slides were incubated with the primary antibody for 1 h, followed by a prediluted secondary antibody (SM802, DAKO) for 10 min and the corresponding Opal fluorophore for 10 min. Microwave treatment was used to remove the antibody complex before the next cycle. After all six cycles, the nuclei were counterstained with DAPI, and the slides were mounted with antifade medium.

Images were acquired at 20× magnification using the TissueFAXS Spectra system with identical acquisition parameters for all TMA cores. Spectral unmixing was performed in the TissueFAXS software using an in-house spectral library generated from single-Opal-stained and DAPI-only control slides.

The unmixed images were analyzed using StrataQuest software (version 7.1.129, TissueGnostics GmbH, Vienna, Austria). Nuclei were segmented according to DAPI, and marker expression was measured within marker-specific nucleus-centered regions. Positivity thresholds were visually calibrated using approximately 30% of randomly selected TMA cores and were then fixed and uniformly applied to all cores. Regions with severe tissue loss, folding, necrosis, out-of-focus imaging, or strong background fluorescence were excluded.

### 2.10 Cell phenotype analysis

Cell subsets were assigned according to marker combinations: CD68+ cells were defined as total macrophages, CD68+CD163+ cells as M2-like macrophages, CD68+CD163- cells as M1-like macrophages, FOXP3+ cells as Tregs, FAP+ cells as fibroblasts, and PANCK+ cells as tumor/epithelial cells.

### 2.11 Spatial distance analysis

Spatial analyses were performed using StrataQuest software (version 7.1.129, TissueGnostics GmbH). The entire evaluable tumor region of one TMA core per patient was used as a single ROI, after excluding areas with insufficient signal quality. Approximately 6,500 segmented cells were analyzed per ROI. No specific edge correction was applied.

For each predefined ordered cell pair, the first and second phenotypes were designated as the reference and target cells, respectively. A directional first nearest-neighbor algorithm was used to calculate the shortest distance between the segmented nuclear boundaries of each reference cell and its nearest target cell. The number of nearest-neighbor pairs within 25 μm and the mean and minimum distances were quantified. The 25 μm cutoff was used to define short-range spatial proximity based on previous multiplex imaging studies using 25–30 μm cellular neighborhoods (29, 30). For tumor-centered infiltration analysis, PanCK-positive tumor cells were used as the reference, and target cells were quantified within 0–10, 10–20, 20–30, and 30–40 μm distance intervals.

### 2.12 Statistical analysis

R software (version 4.2.1) and GraphPad Prism (version 8.0) were used for statistical analysis. Data are expressed as the mean +/- standard error of the mean. Differential expressions was analyzed with limma in R, and p-values were obtained by two-sided Wilcoxon rank-sum test or adjusted for multiple testing when necessary. The chi-square test or Fisher’s exact test was used for categorical variables. Kaplan-Meier curves were compared with the log-rank test. Univariate and multivariate Cox proportional hazards models were used to evaluate prognostic independence. Variables with P < 0.05 in the univariable Cox regression analysis were entered into the multivariable model. The proportional hazards assumption was assessed using Schoenfeld residuals. All tests were two-sided, and p < 0.05 was considered significant.

## 3 Results

### 3.1 STARD3NL expression is upregulated in HCC

**Figure 1:**
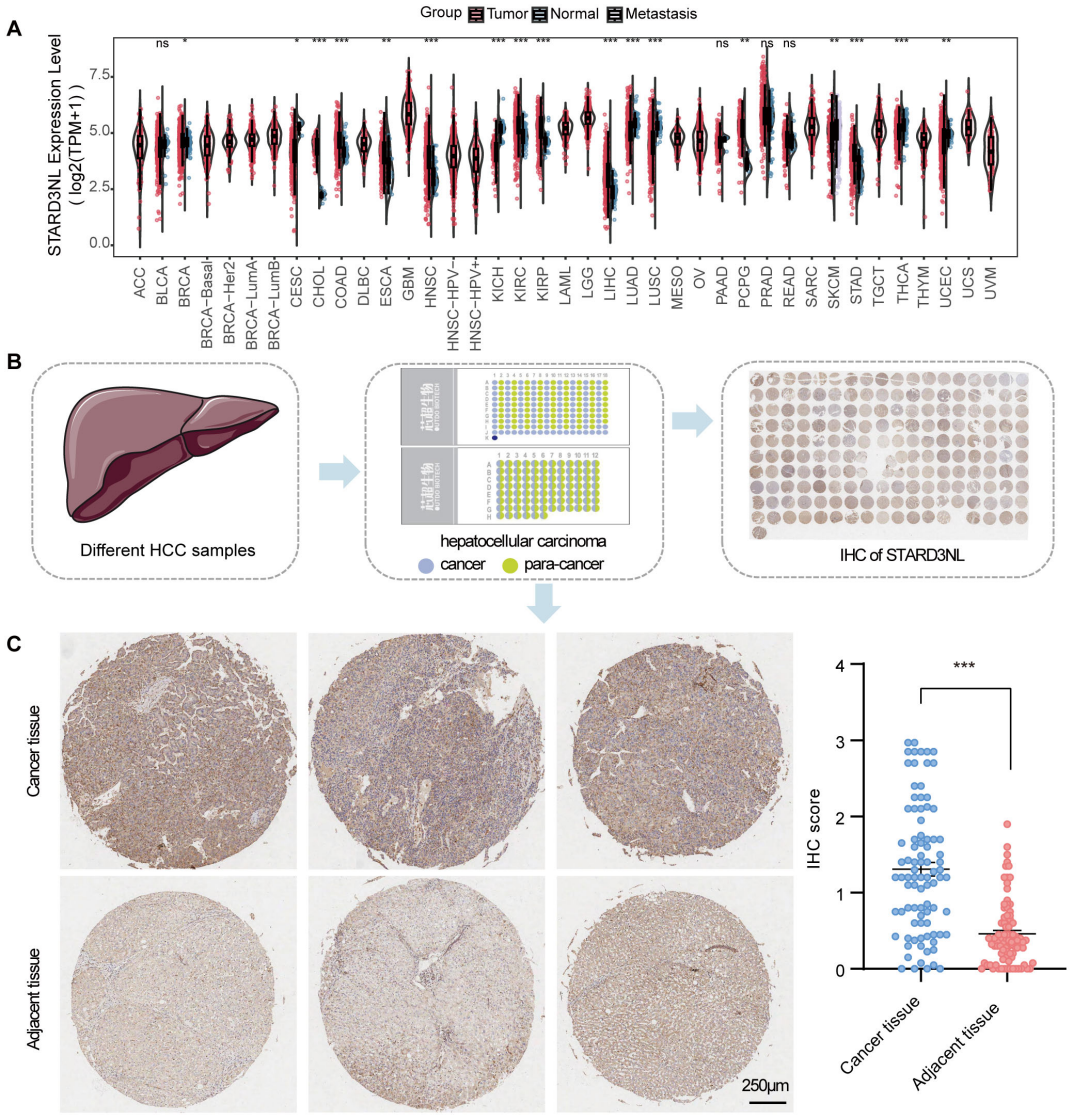
STARD3NL expression analysis and validation in HCC. **(A)** TCGA-based pan-cancer analysis showing STARD3NL mRNA expression across tumor types. ns, not significant; *p <0.05, **p <0.01, ***p <0.001. **(B)** Overview of the experimental design. Two HCC clinical cohorts were used to construct TMAs; one was examined by IHC and the other by mIHC. **(C)** STARD3NL staining is stronger in HCC tumor tissues than in adjacent non-tumor tissues (n = 88). Scale bar: 250 micrometers.

We first examined STARD3NL expression in HCC using TIMER 3.0. STARD3NL was clearly increased in liver cancer samples (**Figure 1A**). We then performed IHC on an HCC TMA containing 88 samples to validate this finding at the protein level (**Figure 1B**). Tumor tissues showed stronger STARD3NL staining than adjacent normal tissues (**Figure 1C**), in agreement with the mRNA-level result.

### 3.2 STARD3NL links to HCC prognosis and clinical features

**Figure 2:**
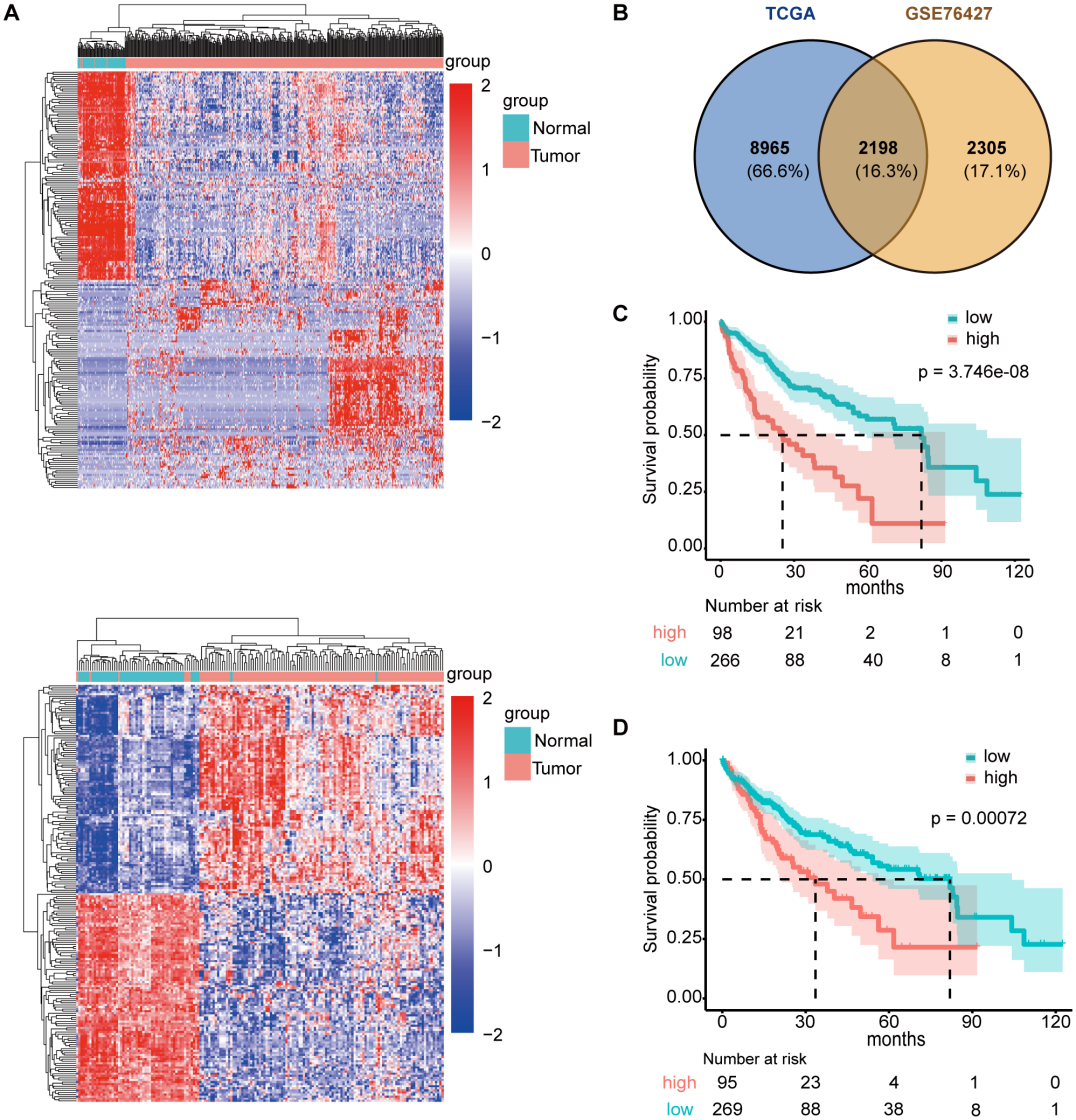
Bioinformatic assessment of the prognostic value of STARD3NL in HCC. **(A)** Hierarchical clustering heatmaps and volcano plots for DEGs identified in TCGA (left) and GSE76427 (right). **(B)** Overlap of upregulated DEGs shared by the TCGA and GSE76427 datasets. **(C)** OS curves stratified by STARD3NL expression in the TCGA cohort. **(D)** Kaplan-Meier Plotter analysis of OS according to STARD3NL expression.

To explore the clinical relevance of STARD3NL, we screened DEGs between tumor and peritumoral tissues in the GEO and TCGA datasets (**Figure 2A**). A total of 2,198 overlapping upregulated genes were obtained (**Figure 2B**). Cox regression analysis identified 609 upregulated DEGs associated with poor prognosis, and STARD3NL was among these high-risk genes (**Supplementary Material 1**). Consistent survival results were obtained from TCGA and the Kaplan-Meier Plotter database (**Figures 2C, D**), supporting STARD3NL as a candidate prognostic marker in HCC.

**Figure 3:**
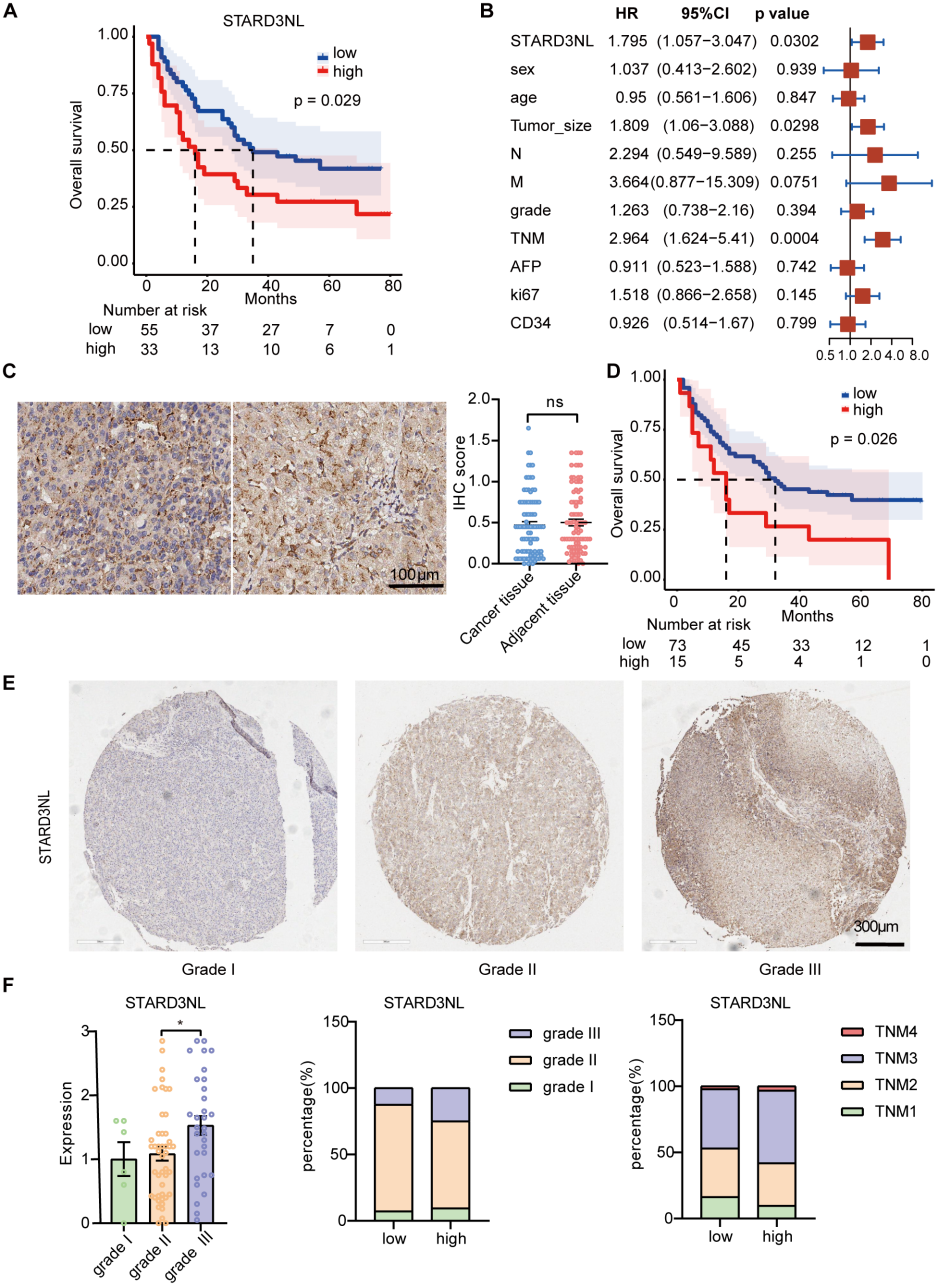
TMA-based validation of STARD3NL associations with OS and clinical features. **(A)** OS curves for patients grouped by STARD3NL expression. High expression was associated with poorer prognosis (p = 0.029). **(B)** Univariable Cox proportional hazards regression analysis of overall survival in the IHC validation cohort. **(C)** Punctate STARD3NL staining did not differ significantly between HCC and adjacent normal tissues. **(D)** OS analysis based on punctate STARD3NL staining. High punctate expression was linked to poorer prognosis (p = 0.026). **(E)** STARD3NL staining intensity increases with pathological grade in HCC. Scale bar: 300 micrometers. **(F)** Distribution of high and low STARD3NL expression across pathological grades and TNM stages in HCC. *P < 0.05.

We further tested this association in the HCC TMA cohort. Patients with high STARD3NL expression had significantly shorter survival than those with low expression (**Figure 3A**). Univariable Cox regression analysis showed that STARD3NL expression, tumor size, and TNM stage were associated with overall survival (**Figure 3B**). These three variables were subsequently entered into the multivariable Cox regression model. After adjustment for tumor size and TNM stage, high STARD3NL expression remained independently associated with poorer overall survival (HR = 2.15, 95% CI: 1.14–4.08, P = 0.0186; **Supplementary Material 2A**). Advanced TNM stage also remained significant (HR = 2.55, 95% CI: 1.26–5.17, P = 0.0096), whereas tumor size was not significant in the multivariable model (HR = 1.37, 95% CI: 0.66–2.84, P = 0.402). No significant violation of the proportional hazards assumption was detected for the individual variables or the overall model (**Supplementary Material 5**).

Previous studies have implicated STARD3NL in membrane contact sites that regulate lipid tethering and lipid balance. Its endosomal localization often appears as punctate staining (20). We therefore evaluated this punctate pattern in our IHC cohort. The level of punctate STARD3NL staining was not significantly different between tumor and adjacent normal tissues (**Figure 3C**). However, patients with stronger punctate STARD3NL expression had poorer OS (**Figure 3D**). In addition, Fisher’s exact test showed that high punctate STARD3NL expression was positively related to advanced pathological grade (p = 0.018; **Supplementary Material 3**).

The association between STARD3NL and clinicopathological features was then analyzed. High STARD3NL expression was more common in tumors with higher pathological grade (**Table 1**). Consistently, increased STARD3NL expression was linked to adverse HCC characteristics, including higher pathological grade and more advanced TNM stage (**Figures 3E, F**).

**Table 1:** Clinical correlation with STARD3NL expression in HLiv-HCC180Sur-04.

Character	Group	Low-STARD3NL n=55	High-STARD3NL n=33	Total n=88	P-value
Sex	Female	49	31	80	0.705
	Male	6	2	8	
Age	≤54	32	15	47	0.276
	>54	23	18	41	
Tumor size	≤7cm	29	20	49	0.502
	>7cm	26	12	38	
TNM	I-II	26	13	39	0.366
	III-IV	23	18	41	
Pathological grade	I-II	40	16	56	0.038
	III	15	17	32	
AFP	≤60	26	15	41	0.651
	>60	22	16	38	
Ki67	≤15	32	15	47	0.101
	>15	15	16	31	
CD34	≤120	24	20	44	0.133
	>120	21	7	28	

### 3.3 STARD3NL expression correlates with immune infiltration

**Figure 4:**
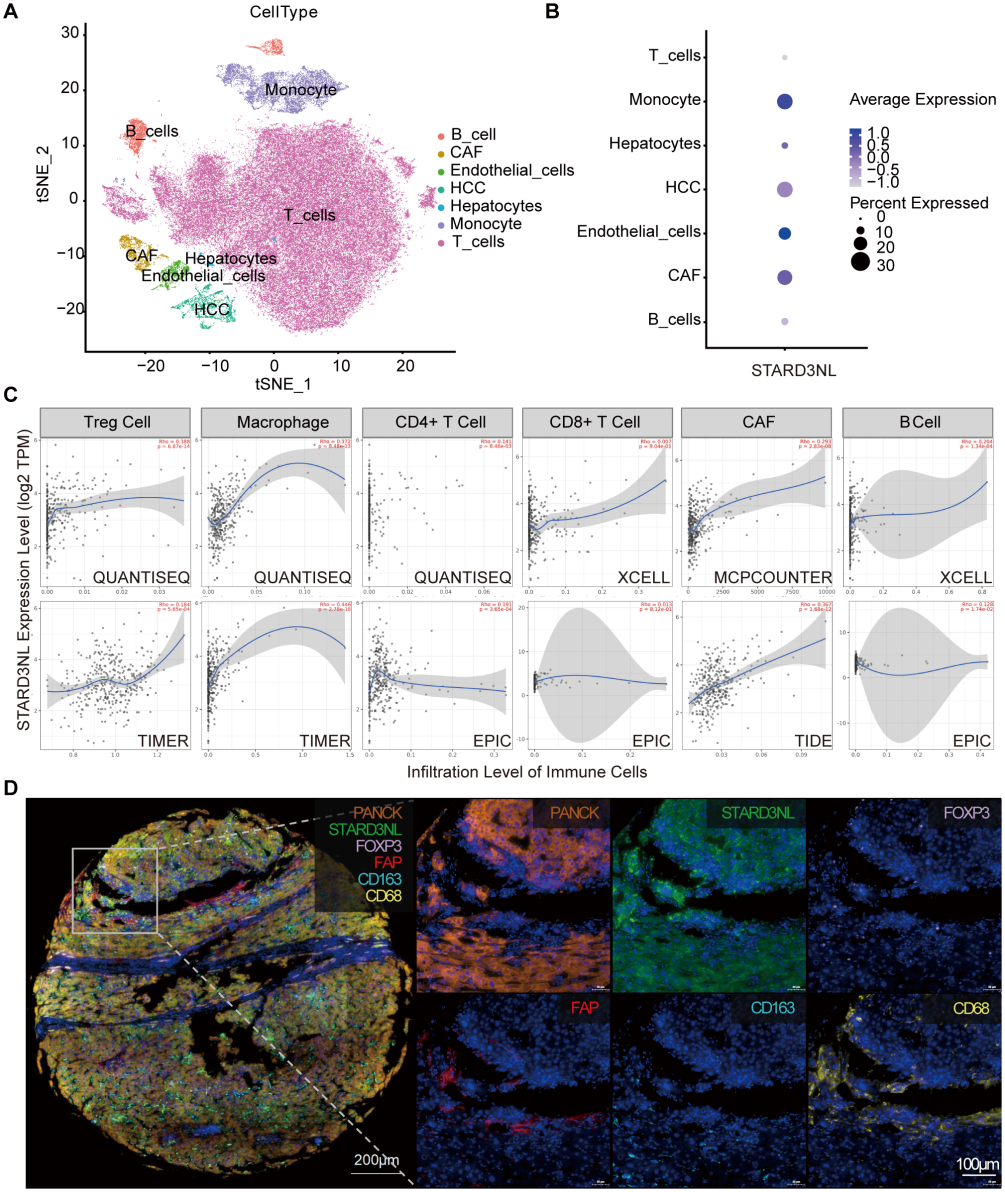
TMA validation showing the relationship between STARD3NL expression and immune infiltration. **(A)** t-SNE plot of 111,437 cells from 34 liver cancer samples in GSE189903, colored by annotated cell type. **(B)** Dot plot of STARD3NL expression in seven cell types. Dot size shows the fraction of expressing cells, and color intensity shows mean expression. **(C)** Immune infiltration analysis of six immune-related populations by TIMER, QUANTISEQ, XCELL, EPIC, MCPCOUNTER and TIDE. **(D)** Representative mIHC image from HCC tissue showing PANCK (orange), STARD3NL (green), FOXP3 (pink), FAP (red), CD163 (cyan), and CD68 (yellow). Scale bar: 250 micrometers.

To determine whether STARD3NL relates to the TME, we analyzed the HCC scRNA-seq dataset GSE189903, which contains 34 samples from GEO. After quality control, normalization, dimensionality reduction, and clustering, cell populations were annotated with SingleR (**Figure 4A**). STARD3NL transcripts were detected in several populations, including monocytes, hepatocytes, malignant hepatocyte-like cells, endothelial cells, and fibroblasts (**Figure 4B**). Immune infiltration analysis also showed that STARD3NL expression correlated with Treg cells, macrophages, CD4+ T cells, CAFs and B cells (**Figure 4C**).

We next used mIHC in a 70-sample HCC TMA cohort to validate the relationship between STARD3NL and immune infiltration. The panel simultaneously detected STARD3NL, FOXP3 for Tregs, FAP for fibroblasts, CD68 for macrophages, CD163 for M2-like macrophages, and PANCK for tumor/epithelial cells (**Figure 4D**). STARD3NL-positive areas largely overlapped with PANCK-positive tumor regions, where immune cells were frequently located nearby. These observations imply that STARD3NL is mainly present in tumor regions and may be linked to local immune cell recruitment or interaction. Fisher’s exact test also showed associations of STARD3NL expression with pathological grade and CTLA4 expression (**Table 2**), supporting a possible connection between STARD3NL and an immunosuppressive HCC microenvironment.

**Table 2:** Clinical correlation with STARD3NL expression in HLivH090Su01.

Character	Group	Low-STARD3NL n=36	High-STARD3NL n=34	Total n=70	P-value
Sex	Female	5	5	10	1
	Male	31	29	60	
Age	≤52	19	18	37	1
	>52	17	16	33	
Tumor size	≤4cm	21	17	38	0.632
	>4cm	15	17	32	
TNM	I	23	22	45	1
	II-III	13	12	25	
Pathological grade	I-II	23	11	34	0.010
	III	13	23	36	
Cirrhosis	Negative	5	3	8	0.710
	Positive	30	31	61	
HBsAg	Negative	10	6	16	0.394
	Positive	25	28	53	
HBcAb	Negative	3	3	6	1
	Positive	30	31	61	
AntiHCV	Negative	33	33	66	1
	Positive	0	1	1	
TB	≤12.6umol/L	18	18	36	1
	>12.6umol/L	17	16	33	
ALT	≤35U/L	17	19	36	0.632
	>35U/L	18	15	33	
ALB	≤4.3g/dl	16	24	40	0.051
	>4.3g/dl	19	10	29	
AFP	≤73ug/L	17	16	33	1
	>73ug/L	18	18	36	
GGT	≤50U/L	18	16	34	0.811
	>50U/L	17	18	35	
PTT	≤11.5	21	15	36	0.232
	>11.5	14	19	33	
PDL1	≤7.5	19	14	33	0.332
	>7.5	15	19	34	
CTLA4	≤125	28	16	44	0.004
	>125	5	17	22	

To further explore the potential pathways through which STARD3NL may be involved in immune regulation, we performed GSEA using the MSigDB C2 canonical pathway collection (**Supplementary Material 4**). TCGA-LIHC samples were stratified into STARD3NL-high and STARD3NL-low groups based on the optimal expression cutoff. GSEA revealed that lipid metabolism-related pathways, including cholesterol biosynthesis pathways in HCC (NES = 1.366, *p* < 0.05), peroxisomal lipid metabolism (NES = 1.931, *p* < 0.001), and fatty acid omega-oxidation (NES = 2.118, p < 0.001), were significantly enriched in STARD3NL-high tumors. In parallel, immune-related pathways, such as immune infiltration in cancer (NES = 1.434, p < 0.05), interleukin-4/interleukin-13 signaling (NES = 1.466, *p* < 0.05), and interleukin-10 signaling (NES = 1.495, *p* < 0.05), were also positively enriched in the high-expression group. These pathway-level findings provide additional molecular context supporting the association between STARD3NL expression and both lipid metabolic reprogramming and immune-regulatory features in the HCC microenvironment.

### 3.4 Macrophages and Tregs are enriched in STARD3NL-high tumors

To assess the relationship between STARD3NL expression and immune composition, we measured cell counts and cell proportions in the tumor compartment, stromal compartment, and whole TME.

**Figure 5:**
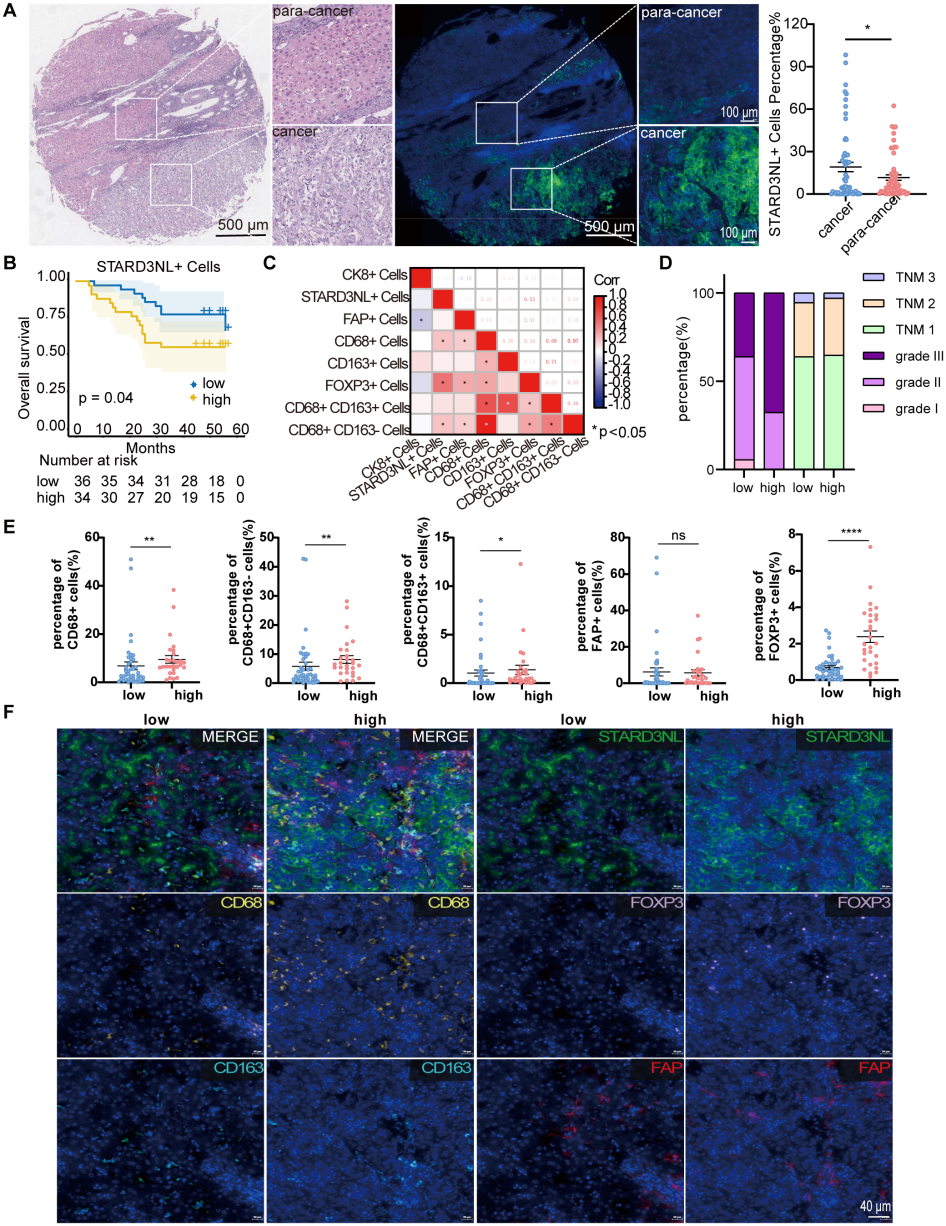
STARD3NL expression and immune cell infiltration in the mIHC cohort. **(A)** STARD3NL expression is enriched in HCC tumor regions compared with adjacent normal regions. Scale bar: 500 micrometers. **(B)** OS curves stratified by STARD3NL expression. High STARD3NL expression was associated with shorter survival (p = 0.04). **(C)** Correlation heatmap of annotated cell populations. STARD3NL+ cell proportion correlated positively with CD68+ and FOXP3+ cell proportions. **(D)** Distribution of STARD3NL-high and STARD3NL-low cases across pathological grades and TNM stages. **(E)** Cell-type proportions in STARD3NL-high and STARD3NL-low tumors. CD68+, CD68+CD163-, and FOXP3+ cells were enriched in the high-expression group. **(F)** Representative staining corresponding to panel **(E)** Green: STARD3NL; yellow: CD68; pink: FOXP3; cyan: CD163; red: FAP. Scale bar: 40 micrometers. ns indicates not significant; *P < 0.05; **P < 0.01; and ****P < 0.0001.

STARD3NL+ cells accounted for a larger fraction of cells in tumor tissues than in adjacent normal tissues (**Figure 5A**). A higher fraction of STARD3NL+ cells was also associated with shorter survival (**Figure 5B**). Correlation analysis across annotated cell populations (**Figure 5C**) showed positive correlations between STARD3NL+ cells and FOXP3+ Tregs (r = 0.53), CD68+ macrophages (r = 0.23), and CD68+CD163- macrophages (r = 0.24). These findings point to the possibility that Tregs and macrophages may be enriched in STARD3NL+ tumors. FOXP3+ Tregs were also positively correlated with CD68+ macrophages (r = 0.34), CD68+CD163- macrophages (r = 0.33), and CD68+CD163+ macrophages (r = 0.23), suggesting possible co-enrichment of immune-suppressive cell types.

The samples were then stratified into STARD3NL-high and STARD3NL-low groups to compare clinical variables and immune composition. Clinically, the high-expression group included more grade III tumors, whereas TNM stage did not differ significantly between the groups (**Figure 5D**). Immunologically, macrophages and Tregs were more abundant in STARD3NL-high tumors, while fibroblast proportions showed no clear between-group difference (**Figures 5E, F**).

**Figure 6:**
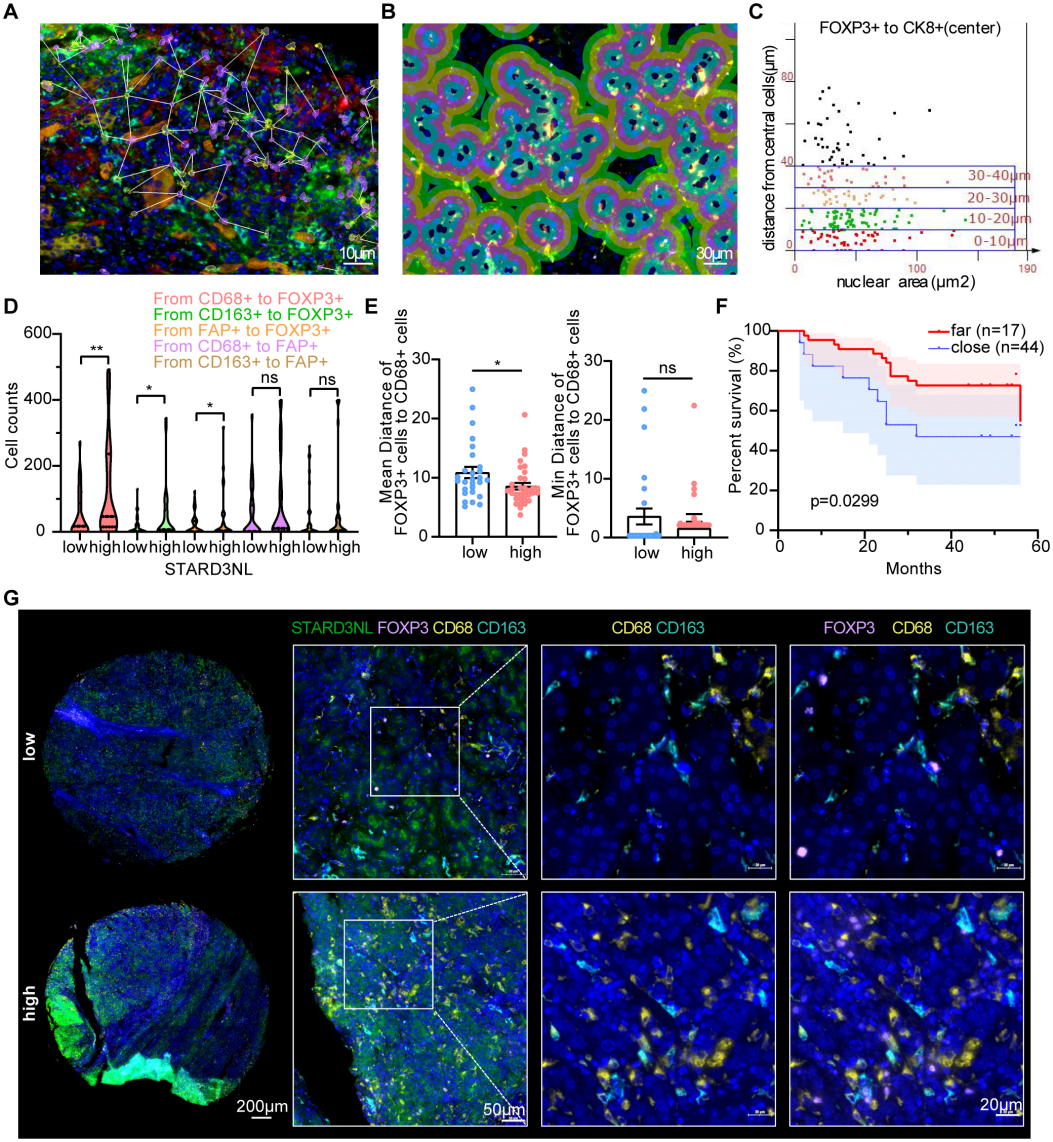
Spatial organization of CD68+ macrophages and FOXP3+ Tregs in STARD3NL-high HCC. **(A)** Example image used for nearest-neighbor distance analysis between CD68+ cells and FOXP3+ cells within 25 micrometers. Yellow circles, CD68+ cells; pink circles, FOXP3+ cells; white lines, intercellular distances. Scale bar: 30 micrometers. **(B)** Schematic of distance-based infiltration analysis from tumor epithelial cells. The 0-10, 10-20, 20-30, and 30–40 micrometer areas are shown in blue, pink, yellow, and green, respectively. Scale bar: 30 micrometers. **(C)** Quantification schematic for panel **(B)** Each dot indicates one cell and was used to calculate total cell number, target cell number, and regional density. **(D)** Neighboring-cell counts for different cell pairs compared between STARD3NL-high and STARD3NL-low groups (n = 70). **(E)** Average and minimum CD68+ cell-FOXP3+ cell distances compared between STARD3NL-high and STARD3NL-low groups (n = 70). **(F)** Survival analysis based on the distance between CD68+ cells and FOXP3+ cells. **(G)** Summary showing that STARD3NL-high tumors contain more CD68+ and FOXP3+ cells and show shorter distances between these populations. ns indicates not significant; *P < 0.05; **P < 0.01.

We further investigated the spatial arrangement of tumor cells, macrophages, and Tregs in tumors with high STARD3NL expression (**Figures 6A–C**). Within 25 micrometers of total macrophages and M2-like macrophages, more neighboring Tregs were detected, and the average distance between these cells was shorter (**Figures 6D, E**). Patients showing this close cell-cell pattern had worse survival (**Figure 6F**). Distance-based infiltration analysis also showed higher Treg counts around tumor cells at most distance intervals, except 30–40 micrometers. In contrast, macrophage counts increased only within 10–20 micrometers from tumor cells (**Supplementary Material 2D**). These results point to close macrophage-Treg organization as a possible contributor to immune suppression (**Figure 6G**).

## 4 Discussion

Research on STARD3NL in cancer is still limited. This protein has mainly been studied in relation to cholesterol trafficking and lipid homeostasis (19, 31). Cholesterol metabolism is increasingly recognized as an important component of tumor biology because it supports membrane biogenesis and oncogenic signaling and also modulates immune cells within the TME (32). In HCC, cholesterol-related metabolic changes have been implicated in tumor initiation and progression (33–35). Although STARD3NL has not been well characterized in HCC, STARD3, which contains a similar N-terminal region, has been implicated in HCC recurrence (36). In this study, integrated analysis of TCGA and GEO data identified STARD3NL as an upregulated high-risk gene in HCC, and this result was validated in clinical samples by IHC and mIHC. Importantly, STARD3NL remained an independent indicator of poor OS.

Our work provides an initial evaluation of the interplay between STARD3NL and the HCC immune microenvironment. Lipid accumulation has been reported to favor M2-like activation of tumor-associated macrophages (21, 37), and these macrophages can promote Treg recruitment or activation, thereby weakening antitumor immune responses (38). Both public data analysis and mIHC results showed that STARD3NL expression was positively associated with macrophage and Treg infiltration. We also observed a shorter spatial distance between macrophages and Tregs in STARD3NL-high tumors. Macrophages and Tregs are central regulators of antitumor immunity. Macrophages can shift between M1-like and M2-like states under the influence of cytokines and chemokines in the TME (39). However, it should be acknowledged that macrophage polarization in the tumor microenvironment exists as a dynamic continuum rather than as two discrete phenotypes (40). The widely used markers CD68 and CD163, while convenient for immunohistochemical detection, are insufficient to fully capture the complexity of macrophage functional states (41). Ideally, a more comprehensive marker panel—including HLA-DR, NOS2, or CD80 for M1-like polarization and CD206 or ARG1 for M2-like polarization—would enable more accurate phenotypic classification (42). In the present study, we emphasized that CD68^+^CD163^+^ and CD68^+^CD163^-^ represent operationally defined marker phenotypes rather than definitive M2 and M1 functional states. Nevertheless, we acknowledge that this approach provides only a simplified view of macrophage heterogeneity, and our interpretation of macrophage polarization should be viewed with this limitation in mind.

Several recent studies have provided mechanistic insights into lipid-associated immune regulation in the TME that raise the possibility of being relevant to our findings. First, lipid droplet-rich macrophages (LLMs) in HCC have been shown to recruit Tregs through the CCL20/CCR6 axis, establishing an immune-suppressive niche that promotes tumor progression (22). Second, tumor-associated macrophage-derived platelet factor (PF4) can enhance TH1-like Treg polarization and suppress tumor-reactive T cells (43). Third, CSF1 signaling plays a central role in maintaining the survival and immunosuppressive function of tumor-associated macrophages, and lipid metabolic reprogramming has been implicated in CSF1-driven macrophage polarization (12). Fourth, beyond serving as energy stores, lipid droplets are increasingly recognized as dynamic organelles that regulate macrophage inflammatory responses and antigen presentation (17). Given that STARD3NL functions in cholesterol transport and lipid homeostasis at ER-endosome contact sites (19, 20), it is tempting to hypothesize that STARD3NL may influence the TME through lipid metabolism-dependent pathways—for instance, by modulating cholesterol availability or oxysterol signaling in tumor cells or immune cells, thereby affecting chemokine secretion (such as CCL20) or macrophage polarization states. Therefore, the observed enrichment of macrophages and Tregs, together with their closer spatial proximity in STARD3NL-high tumors, is consistent with an immune-suppressive microenvironment. The present study did not directly test whether STARD3NL regulates macrophage polarization or Treg recruitment, and our data should be interpreted as correlative evidence rather than causation. Future studies using perturbation experiments, co-culture systems, and lipidomic profiling will be needed to clarify these mechanisms.

Several limitations should be noted. Although the TMA cohort was suitable for the present analyses, tissue microarrays sample only limited tumor regions, so intratumoral heterogeneity may have been underestimated. Validation in larger independent cohorts is needed. Most importantly, the present study is observational and correlative in nature, and our findings demonstrate significant associations between STARD3NL expression and an immune-suppressive cell landscape including increased macrophage and Treg infiltration and reduced macrophage-Treg spatial distance. Our future studies are warranted to establish causality and to define whether and how STARD3NL directly influences immune cell recruitment or function. Overall, our data indicates that STARD3NL is a potential prognostic biomarker associated with an immune-suppressive TME phenotype in HCC.

## Data Availability

The datasets presented in this study can be found in online repositories. The names of the repository/repositories and accession number(s) can be found in the article/**Supplementary Material**.

## Supplementary material

Supplementary Material 1 Differentially expressed and prognosis-associated genes identified from the TCGA-LIHC and GSE76427 datasets. The “diffgene” worksheet lists 2,198 genes that were commonly upregulated in HCC tissues in both datasets, together with their differential-expression statistics. The “prognosis” worksheet lists 609 upregulated genes associated with poorer overall survival according to Cox regression analysis, together with their P values and hazard ratios.

Supplementary Material 2 Additional analyses of the prognostic significance, single-cell expression pattern, and spatial immune-cell distribution associated with STARD3NL. **(A)** Forest plot of the multivariable Cox regression model including STARD3NL expression, tumor size, and TNM stage. After adjustment for tumor size and TNM stage, high STARD3NL expression remained independently associated with poorer overall survival (HR = 2.15, 95% CI: 1.14–4.08, P = 0.0186). **(B)** t-SNE feature plot showing the distribution of STARD3NL expression across 111,437 cells from 34 HCC samples in the GSE189903 dataset. **(C)** Violin plots showing STARD3NL expression across seven annotated cell populations. **(D)** Numbers of FOXP3+, CD68+, CD68+CD163+, and CD68+CD163− cells located within 0–10, 10–20, 20–30, and 30–40 μm of PanCK+ tumor cells in the STARD3NL-low and STARD3NL-high groups. Each dot represents one tissue sample, and error bars indicate the mean ± SEM. Statistical comparisons were performed using the two-sided Wilcoxon rank-sum test. ns, not significant; *P < 0.05; **P < 0.01.

Supplementary Material 3 Association between punctate STARD3NL expression and clinicopathological characteristics in the HCC cohort. Patients were classified into STARD3NL-low and STARD3NL-high groups according to punctate STARD3NL staining. Associations with clinicopathological variables were evaluated using Fisher’s exact test. The φ coefficient indicates the strength and direction of association. Cases with unavailable clinical information were excluded from the corresponding analysis.

Supplementary Material 4 Gene set enrichment analysis of pathways associated with STARD3NL expression in TCGA-LIHC. TCGA-LIHC samples were classified into STARD3NL-high and STARD3NL-low groups according to the optimal expression cutoff. The displayed pathways were positively enriched in the STARD3NL-high group: **(A)** cholesterol biosynthesis pathway in hepatocytes (NES = 1.366, P = 0.031); **(B)** peroxisomal lipid metabolism (NES = 1.931, P = 7.507 × 10−4); **(C)** fatty acid omega-oxidation (NES = 2.118, P = 2.751 × 10−7); **(D)** immune infiltration in cancer (NES = 1.434, P = 0.028); **(E)** interleukin-10 signaling (NES = 1.495, P = 0.030); and **(F)** interleukin-4 and interleukin-13 signaling (NES = 1.466, P = 0.0147). NES, normalized enrichment score.

Supplementary Material 5 Assessment of the proportional hazards assumption for the multivariable Cox regression model. The proportional hazards assumption was evaluated using Schoenfeld residuals for STARD3NL expression, tumor size, and TNM stage. No significant violation was detected for STARD3NL expression (P = 0.300), tumor size (P = 0.653), TNM stage (P = 0.699), or the overall model (global test, P = 0.614).
